# The Contribution of Native Protein Complexes to Targeted Protein Degradation

**DOI:** 10.1021/acschembio.6c00098

**Published:** 2026-04-09

**Authors:** Lorraine Glennie, Nicole M. Curnutt, Gajanan Sathe, Brune Le Chatelier, Freya Goff, Jin-Feng Zhao, Tyrell Cartwright, Karen Dunbar, Nicola T. Wood, Thomas J. Macartney, Christina M. Woo, Gopal P. Sapkota

**Affiliations:** † Medical Research Council Phosphorylation & Ubiquitylation Unit (MRC-PPU), School of Life Sciences, 3042 University of Dundee, Dow Street, Dundee, Scotland DD1 5EH, U.K.; ‡ Department of Chemistry and Chemical Biology, 1812 Harvard University, Cambridge, Massachusetts 02138, United States; § Broad Institute of MIT and Harvard, Cambridge, Massachusetts 02142, United States

## Abstract

Targeted protein degradation (TPD) destroys proteins of interest (POIs) by hijacking the cellular proteolytic machinery. Most proteins in cells exist and function as part of multiprotein or macromolecular complexes, thereby allowing a single protein to control multiple biological processes. Therefore, when a small molecule degrader induces the proximity between an E3 ligase and the POI, the macromolecular context of the POI potentially influences the degradation outcomes of the POI and of the complex components. Here, we explore the degradation of the eight CK1α-SACK1 (formerly known as FAM83A-H) complexes initiated by molecular glue degraders primarily designed to target Ser/Thr kinase CK1α. We demonstrate that lenalidomide-derived degraders DEG-77 and SJ3149, which selectively target the CK1α isoform, codegrade multiple SACK1 proteins. We show that the degradation of SACK1 proteins by DEG-77 and SJ3149 requires CK1α, the CUL4A^CRBN^ E3 ligase complex, and the proteasome. In cells derived from palmoplantar keratoderma patients harboring the CK1α-binding-deficient SACK1G^R265P^ mutation, DEG-77 targets CK1α and mitotic SACK1D but not SACK1G^R265P^, highlighting the requirement for CK1α-SACK1 interaction to achieve codegradation. Our study underscores the importance of POI context in TPD and reinforces the potential for selectively targeting specific protein complexes for degradation.

## Graphical abstract

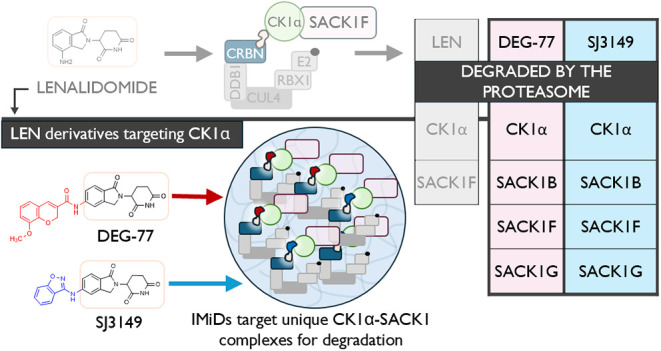

## Introduction

A protein’s function within a cell is shaped by its subcellular localization, interacting partners, post-translational modifications, and turnover dynamics.^1,2^ As such, protein–protein interactions and multiprotein complexes constitute the fundamental functional modules that orchestrate cellular signaling and homeostasis.^3,4^ A single protein can participate in diverse cellular processes by forming unique macromolecular complexes, depending on the context. The Ser/Thr protein kinase CK1α, a prototypic member of the CK1 kinase family, exemplifies this principle. CK1α regulates numerous cellular processes, including WNT signaling, cell division, apoptosis, calcium signaling, and cellular responses to DNA damage.^5–9^ While the mechanisms underlying its broad activity are still being elucidated, the eight proteins of the FAM83 family (A-H; hereafter referred to as SACK1A-H) are recognized as key regulators of CK1α and other CK1 isoforms.^10–13^ They interact with CK1α through the conserved Scaffold Anchor of CK1 (SACK1) domain (formerly known as DUF1669), dictating its subcellular localization and potentially its substrate repertoire.^11,13^ SACK1D recruits CK1α to the mitotic spindle to orchestrate proper spindle alignment and timely cell division.^6^ The CK1α-SACK1F complex at the plasma membrane and CK1α-SACK1G complex primarily in the cytoplasm drive the activation of canonical WNT signaling.^10,12^ Indeed, loss of CK1α-SACK1G interaction, leading to inhibition of canonical WNT signaling, underlies the pathogenesis of palmoplantar keratoderma (PPK) caused by autosomal recessive *FAM83G* mutations in both humans^14–16^ and dogs.^17^ Four reported missense *FAM83G* mutations in PPK, which lead to A34E, R52P, F58I, and R265P substitutions on the SACK1G protein, all lie within the SACK1 domain and disrupt CK1α binding.^14–16,18^

Targeted protein degradation (TPD), via proteolysis-targeting chimeras (PROTACs), molecular glue degraders (MGDs), or other modalities, is emerging as a powerful approach for investigating protein function in cells and developing targeted therapeutics.^19–23^ PROTACs and MGDs function by inducing spatial proximity between an E3 ubiquitin (Ub) ligase and the target protein of interest (POI), thereby facilitating POI ubiquitination and subsequent proteasomal degradation.^24–26^ Excitingly, several PROTACs have progressed to human clinical trials, whereas some MGDs based on immunomodulatory imide drugs (IMiDs), such as thalidomide, lenalidomide, and pomalidomide, are already in clinical use against multiple myeloma and other indications.^27–29^ IMiDs act as MGDs by binding to the CUL4A E3 ligase substrate receptor CRBN and facilitate the recruitment of neo-substrates, which then undergo ubiquitination and proteasomal degradation.^24,25,30,31^ Naturally, CRBN is thought to specifically recognize proteins with a C-terminal cyclic imide degron, which arises from the cyclization of glutamine or asparagine residues.^32^ Many neo-substrates recruited to CRBN by IMiD-based MGDs contain C2H2 zinc finger domains, such as transcription factors IKZF1 and IKZF3.^25,33,34^ Proteins like CK1α are also recruited by lenalidomide as a neo-substrate through its exposed β-hairpin motif in the N-lobe of the kinase domain.^24,35^ Degradation of CK1α is thought to contribute to the clinical efficacy of lenalidomide against myelodysplastic syndromes (MDS) caused by the deletion of chromosome 5q that results in the loss of one *CSNK1A1* (CK1α) allele.^24^ Successful clinical applications of IMiDs have rejuvenated profound interest in developing even more potent and selective CRBN ligands that target C2H2 zinc-finger-containing proteins as well as those that contain exposed β-hairpin motifs similar to that of CK1α.^33,34,36^

Independent efforts to improve selectivity and potency of CK1α degradation with lenalidomide derivatives have led to a novel series of potent CK1α degraders. The DEG series of compounds derived from lenalidomide resulted in DEG-77 and DEG-35 as potent dual CK1α and IKZF2 degraders with efficacy against acute myeloid leukemia.^37,38^ DEG-77 also displayed antiproliferative activity against diffuse large B cell lymphoma cell line OCI-LY3 and the ovarian cancer cell line A2780.^38^ Independently developed lenalidomide derivatives SJ7095, SJ0040, and SJ3149 are also potent CK1α degraders.^39,40^ While SJ7095 degraded CK1α, IKZF1, and IKZF3, and caused cytotoxicity against acute myeloid leukemia MOLM-13 cells, SJ0040 and SJ3149 improved selectivity against CK1α but still maintained antiproliferative effects.^40^

TPD in cells is primarily driven by the generation of a productive POI-degrader-E3 ternary complex. Beyond chemical properties of the degrader, there are many cellular factors that can influence the formation of this ternary complex and thus the specificity and potency of the degrader.^41–43^ For example, subcellular localization of the POI and/or E3 ligase, the choice of the E3 ligase being recruited, and the abundance and activity of the E3 ligase can all influence the extent and kinetics of TPD.^42,44,45^ Other cellular factors such as degrader uptake and retention, degrader metabolism, and protein turnover rates can also affect TPD.^41^ One crucial, and often overlooked, factor in TPD that can influence the formation of the POI-degrader-E3 ternary complex is the inherent POI macromolecular context that defines its interactions with other macromolecules, localization, and activity. Based on this POI context, the extent and kinetics of POI degradation, as well as potential degradation of other proteins in complex with the POI, often referred to as “*bystander degradation’*” or “*collateral damage*”, could be affected.

Previously, we showed that lenalidomide, which only modestly degrades CK1α in cells, leads to complete codegradation of SACK1F but not other SACK1 proteins.^31^ Given recent reports of potent and selective CK1α degraders^37–40^ and our findings that CK1α in cells exists within a unique subcellular location dictated by its interaction with one of eight SACK1A-H proteins,^11^ we postulated that these degraders could also influence specific or multiple CK1α-SACK1A-H complexes. Here, we explore the effects of DEG^38^ and SJ^40^ series of potent CK1α degraders on the stability of SACK1A-H proteins.

## Results

### Assessment of Endogenous CK1α-SACK1 Complexes in Multiple Human Cell Lines

The eight SACK1A-H proteins are known to direct the localization of the Ser/Thr kinase CK1α to distinct subcellular compartments (Figure 1A).^11,13^ We monitored the expression of CK1α and different SACK1A-H proteins and their interactions at the endogenous level in several human cancer cell lines. In DLD1 colorectal cancer cells, the expression of CK1α, SACK1B, SACK1D, SACK1F, SACK1G, and SACK1H was detected by immunoblotting (Figure 1B). In U2OS osteosarcoma cells, CK1α, SACK1D, SACK1G, and SACK1H were detected, while in A549 lung adenocarcinoma cells, only CK1α, SACK1G, and SACK1H were detected (Figure 1B). In TOV-21G ovarian cancer cells, CK1α, SACK1B, SACK1D, SACK1G, and SACK1H were detected (Figure 1C). With our existing antibodies, we were unable to detect SACK1A, SACK1C, and SACK1E at the endogenous level in any of the cell lines. Immunoprecipitates (IPs) of endogenous CK1α with anti-CK1α antibody coprecipitated SACK1B, SACK1F, SACK1G and SACK1H from DLD1 extracts, SACK1G and SACK1H from U2OS and A549 extracts, and SACK1B, SACK1G and SACK1H from TOV-21G extracts (Figure 1B). No SACK1D was coprecipitated in anti-CK1α IPs from any of the extracts, which were harvested under asynchronous conditions (Figure 1B,C). This is consistent with our previous findings that SACK1D-CK1α interaction occurs only during mitosis and not in other stages of the cell cycle.^6^ As expected, IPs with IgG controls did not precipitate CK1α or any SACK1 protein (Figure 1B,C). Using these cells, we sought to test the impact of CK1α degraders on different SACK1 proteins.

**Figure 1:**
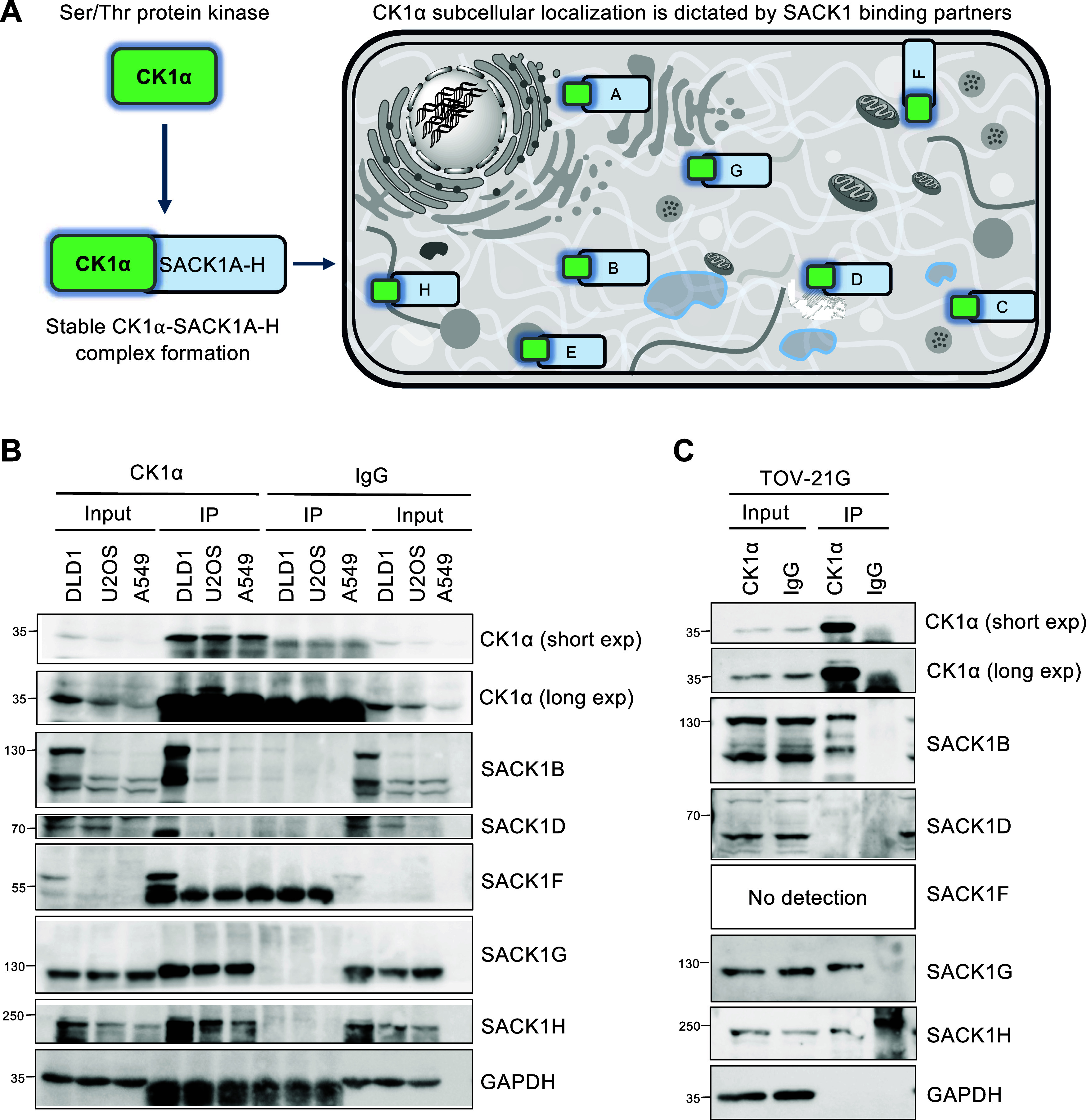
SACK1 proteins form complexes with CK1α at the endogenous level in DLD1, U2OS, A549, and TOV-21G cell lines. (A) Depiction of subcellular distribution of serine/threonine protein kinase CK1α and interacting SACK1 proteins. SACK1A-H direct CK1α and other CK1 isoforms to distinct subcellular compartments to regulate CK1 biology. (B, C) DLD1, U2OS, A549 (B), and TOV-21G (C) cells were lysed, and extracts were normalized to 2 mg total protein and subjected to anti-CK1α IPs. IgG IPs were used as negative controls. Input extracts and IPs were resolved by SDS-PAGE, transferred to PVDF membrane, and analyzed by immunoblotting using the indicated antibodies.

### DEG Series of CK1α Degraders Reduce Levels of Several SACK1 Proteins

Given that we detected CK1α and most SACK1 proteins in DLD1 cells, we treated these cells with the DEG series^37,38^ of CK1α degraders (Figure S1). DEG compounds (DEG–35, −48, −52, −54, −61, −64, and −77) (Figure S1) were selected due to their range of efficacy of CK1α degradation (low nanomolar to micromolar DC_50, CK1α_) and structural diversity within the DEG series, which we reasoned may lead to differences in destabilization of specific CK1α-SACK1 complexes upon compound treatment. These compounds were added to cells at a concentration of 100 nM for 24 h (Figure 2A). CK1α, SACK1F, and SACK1G were robustly depleted by DEG-35 and DEG-77 with a > 90% reduction in observed protein abundance compared to DMSO-treated control (Figure 2A). DEG-52 and DEG-64 led to modest CK1α and SACK1G depletion but almost complete removal of SACK1F (Figure 2A). DEG-54, DEG-48, and DEG-61 led to only a slight decrease in the level of CK1α and SACK1G but a robust decrease in SACK1F (Figure 2A). SACK1D interacts with CK1α only in mitosis, and this results in its hyperphosphorylation, causing a mobility shift of ∼25 kDa in immunoblot assays.^11^ In DMSO-treated asynchronous DLD1 extracts, we detected two differentially migrating SACK1D signals, an intense lower (∼75 kDa) band and a less intense upper (∼100 kDa) band, suggesting a proportion of mitotic cells under our experimental conditions. The lower band represents the unphosphorylated SACK1D protein that does not interact with CK1α, whereas the upper band represents the mitotic hyperphosphorylated SACK1D bound to CK1α.^6^ DEG-35, DEG-54, DEG-52, DEG-61, and DEG-77 led to a substantial reduction in levels of the 100 kDa hyperphosphorylated SACK1D without affecting the levels of the 75 kDa unphosphorylated SACK1D, whereas DEG-64 resulted in reduction in levels of both pools (Figure 2A). There was only a modest reduction in levels of SACK1B by DEG-35, DEG-54, DEG-52, DEG-64, and DEG-77, but no changes by other DEG compounds (Figure 2A). SACK1H levels did not change substantially by any of the DEG compounds (Figure 2A). While SACK1D, SACK1F, and SACK1G bind selectively to CK1α, SACK1B and SACK1H also bind to CK1δ and CK1ε, which are not targeted for degradation by IMiD compounds.

**Figure 2:**
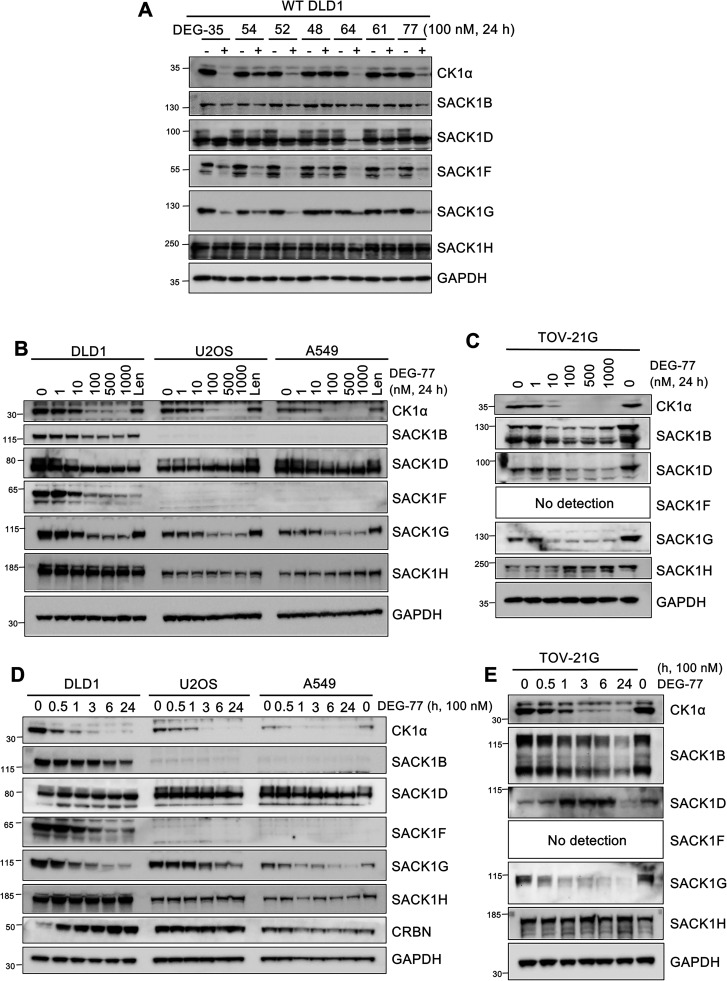
DEG series of CK1α degraders lead to codepletion of CK1α-SACK1 complexes to varying degrees. (A) Wild-type DLD1 cells were incubated with DMSO or 100 nM of each DEG compound (−35, −54, −52, −48, −64, −61, −77) as indicated for 24 h before cell lysis. Extracts were resolved by SDS-PAGE, transferred to PVDF membrane, and immunoblots were incubated with the indicated antibodies. (B) Wild-type DLD1, U2OS, and A549 cell lines were incubated with increasing concentrations of DEG-77 (1–1000 nM) or 10 μM lenalidomide for 24 h before cell lysis. 20 μg of extract protein was resolved by SDS-PAGE and transferred to PVDF membranes for immunoblotting analysis using the indicated antibodies. (C) As in (B), except TOV-21G cells were cultured for 24 h with increasing concentrations of DEG-77 (1–1000 nM) before cell lysis. (D) As in (B), except wild-type DLD1, U2OS, and A549 cells were incubated with 100 nM DEG-77 for different time periods (0.5–24 h) before cell lysis. (E) As in (C), except TOV-21G cells were incubated with 100 nM DEG-77 for different time periods (0.5–24 h) before cell lysis. All blots are representative of at least 3 biological replicates.

Next, to determine if DEG-77 codepletes CK1α and interacting SACK1B, SACK1D, SACK1F, SACK1G, and SACK1H proteins concurrently in multiple cells, DLD1, U2OS, A549, and TOV-21G cells were treated with an increasing concentration of DEG-77 (1–1000 nM) for 24 h (Figure 2B,C). The parental compound lenalidomide (10 μM), from which DEG-77 was derived, was also employed as a control. DEG-77 induced a dose-dependent codepletion of CK1α, SACK1B, hyperphosphorylated SACK1D, SACK1F, and SACK1G but not SACK1H compared to DMSO control in all cells that expressed these proteins, with a robust reduction observed at concentrations of 100 nM or over (Figure 2B,C). Lenalidomide caused a minimal degradation of CK1α and no decrease in levels of SACK1B, SACK1D, SACK1G, and SACK1H, but a robust decrease in SACK1F levels compared to DMSO control (Figure 2B). When cells were treated with 100 nM DEG-77 over a course of 24 h, a time-dependent reduction in levels of CK1α, SACK1B, hyperphosphorylated SACK1D, SACK1F, and SACK1G but not SACK1H was observed (Figure 2D,E). A reduction in levels of CK1α was observed as early as 30 min following DEG-77 treatment, but a robust depletion was observed after 1 h, with peak degradation observed at 3 h, which was sustained for 24 h (Figure 2D,E). The decreased level of SACK1B in DLD1 and TOV-21G cells, hyperphosphorylated SACK1D in DLD1, U2OS, and A549 cells, SACK1F in DLD1 cells, and SACK1G in all cells closely mirrored the degradation kinetics of CK1α (Figure 2D,E).

In TOV-21G cells, no substantial changes in *CSNK1A1* and *SACK1B* transcripts were observed upon DEG-77 treatment compared to DMSO control, while a slight increase in *SACK1G* transcripts and a larger increase in *SACK1H* transcripts were observed with DEG-77 treatment (Figure S3A), suggesting that the reduction in levels of these proteins caused by DEG-77 cannot be due to a decrease in transcription. However, a robust reduction in *SACK1D* transcripts was observed upon DEG-77 treatment compared to DMSO control (Figure S3A). As *SACK1D* gene expression is regulated by the cell cycle,^6^ the reduction in *SACK1D* transcript by DEG-77 can be attributed to its inhibition of the cell cycle.^37^ Since SACK1A, SACK1C, SACK1E, and SACK1F protein expression was absent or undetectable in TOV-21G cells (Figure 2D,E), we monitored whether their transcripts were present at all. To do this, we undertook qPCR analysis in the presence or absence of reverse transcriptase (RT) enzyme. For active gene transcripts, inclusion of the RT enzyme leads to a much lower normalized cycle quantification (Δ*Cq*) value. Absence of “active” transcript is reflected by little to no difference in Δ*Cq* values between the two conditions. Employed as a positive control, the Δ*Cq* values for *SACK1G* show a significant and substantial reduction with the addition of RT enzyme. In both cell lines, we observed little to no change in Δ*Cq* values for *SACK1A*, *SACK1C*, *SACK1E*, and *SACK1F* in the presence or absence of RT enzyme, suggesting that these genes are transcriptionally inactive in these cells, providing a potential explanation as to why these proteins were not detected by Western blot (Figure S3B).

### SJ Series of CK1α Degraders Reduce Levels of Several SACK1 Proteins

Similar to the DEG series of compounds above, we next investigated the SJ series of CK1α degraders^39,40^ (Figure S1) for their ability to codeplete SACK1 proteins. We treated DLD1 cells with increasing concentrations from 1 to 1000 nM of SJ7095, SJ0040, and SJ3149 for 4 h (Figure 3A). All compounds led to a dose-dependent decrease in levels of CK1α, SACK1B, SACK1F, and SACK1G, with reduction observed at doses over 100 nM (Figure 3A). SJ7095 was the least potent CK1α degrader, with reduced CK1α levels observed at 100 nM and optimal degradation occurring at 1–10 μM (Figure 3A). SJ0040 and SJ3149 were both potent CK1α degraders, with modest degradation observed at 10 nM and almost complete CK1α degradation observed at concentrations over 100 nM (Figure 3A). Although there was an observed difference in CK1α degradation efficiency between SJ7095, SJ0040, and SJ3149, all of the compounds at increasing concentration displayed similar reduced levels for SACK1B, SACK1F, and SACK1G (Figure 3A). The improved potency of CK1α degradation by SJ0040 and SJ3149 in comparison to SJ7095 is potentially due to their stabilized interaction with CK1α within the CK1α-IMiD-CRBN complex.^40^

**Figure 3:**
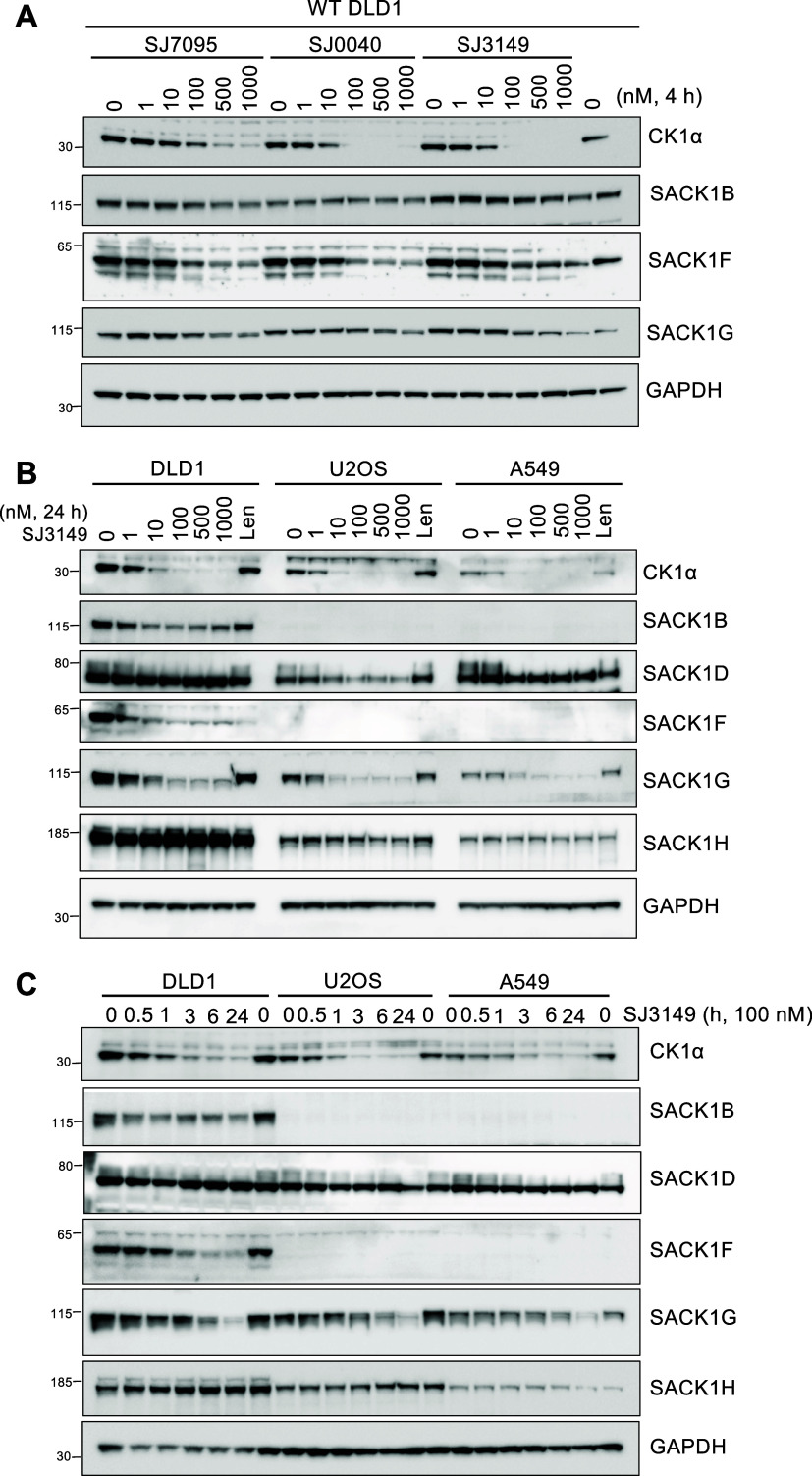
SJ series of CK1α degraders lead to codepletion of CK1α-SACK1 complexes. (A) Wild-type DLD1 cells were treated with SJ compounds (SJ7095, SJ0040, SJ3149) at increasing concentrations from 1 to 1000 nM for 4 h before cell lysis. (B) Wild-type DLD1, U2OS, and A549 cell lines were incubated with increasing concentrations of SJ3149 (1–1000 nM) or 10 μM lenalidomide for 24 h before cell lysis. 20 μg of extract protein was resolved by SDS-PAGE and transferred to PVDF membranes for immunoblotting analysis using the indicated antibodies. (C) As in (B) except wild-type DLD1, U2OS, and A549 cells were incubated with 100 nM SJ3149 for different time periods (0.5–24 h) as indicated before cell lysis. All blots are representative of at least 3 biological replicates.

Next, we treated DLD1, U2OS and A549 cells with increasing doses (1–1000 nM) of SJ3149 for 24 h (Figure 3B). Similar patterns of codepletion in levels of CK1α, SACK1B (in DLD1 cells), hyperphosphorylated SACK1D, SACK1F (in DLD1 cells), and SACK1G were observed compared to DMSO control (Figure 3B). Some depletion was observed with 10 nM SJ3149, with maximal depletion observed at higher (100–1000 nM) concentrations (Figure 3B). No substantial depletion in levels of SACK1H was observed in any cells (Figure 3B). A time-course treatment of 100 nM SJ3149 over 24 h revealed a time-dependent codepletion in levels of CK1α, SACK1B (in DLD1 cells), hyperphosphorylated SACK1D, SACK1F (in DLD1 cells), and SACK1G, with some depletion observed as early as 30 min following SJ3149, and maximal depletion achieved after 3–6 h, which was sustained until 24 h (Figure 3C). The kinetics and potency of codegradation of CK1α and the interacting SACK1 proteins by SJ3149 and DEG-77 were similar (Figures 2 and 3).

### Comparison of CK1α Degraders for Their Ability to Codeplete SACK1 Proteins

Given the differences in codepletion of CK1α and different interacting SACK1 proteins between lenalidomide, DEG-48, DEG-77, SJ7095, and SJ3149, we assessed these CK1α degraders together with a previously reported CK1α and CDK7/9 degrader BTX161 ^46^ to compare how specific CK1α-SACK1 complexes were codepleted by each. DLD1 cells were treated with lenalidomide (10 μM), BTX161 (10 μM), DEG-48 (100 nM), DEG-77 (100 nM), SJ7095 (1 μM), and SJ3149 (100 nM) or DMSO control for 24 h (Figure 4A,B). BTX161, DEG-77, and SJ3149 caused a robust degradation of CK1α, while lenalidomide and SJ7095 caused only a moderate degradation and DEG-48 did not cause any CK1α degradation (Figure 4A,B). For potent degraders, DEG-77 and SJ3149 caused robust codepletion of SACK1B, SACK1F, and SACK1G (Figure 4A,B). BTX161 and lenalidomide only robustly codepleted SACK1F but no other SACK1 protein tested (Figure 4A,B). SJ7095 caused a robust depletion of SACK1F but only a moderate depletion of SACK1G and no degradation of SACK1B and SACK1H (Figure 4A,B). These findings suggest that despite all IMiD compounds engaging CRBN, the specific nature by which each degrader recruits specific CK1α-SACK1 complexes to the CUL4A^CRBN^ E3 ligase machinery potentially dictates how efficiently each complex can be targeted for degradation.

**Figure 4:**
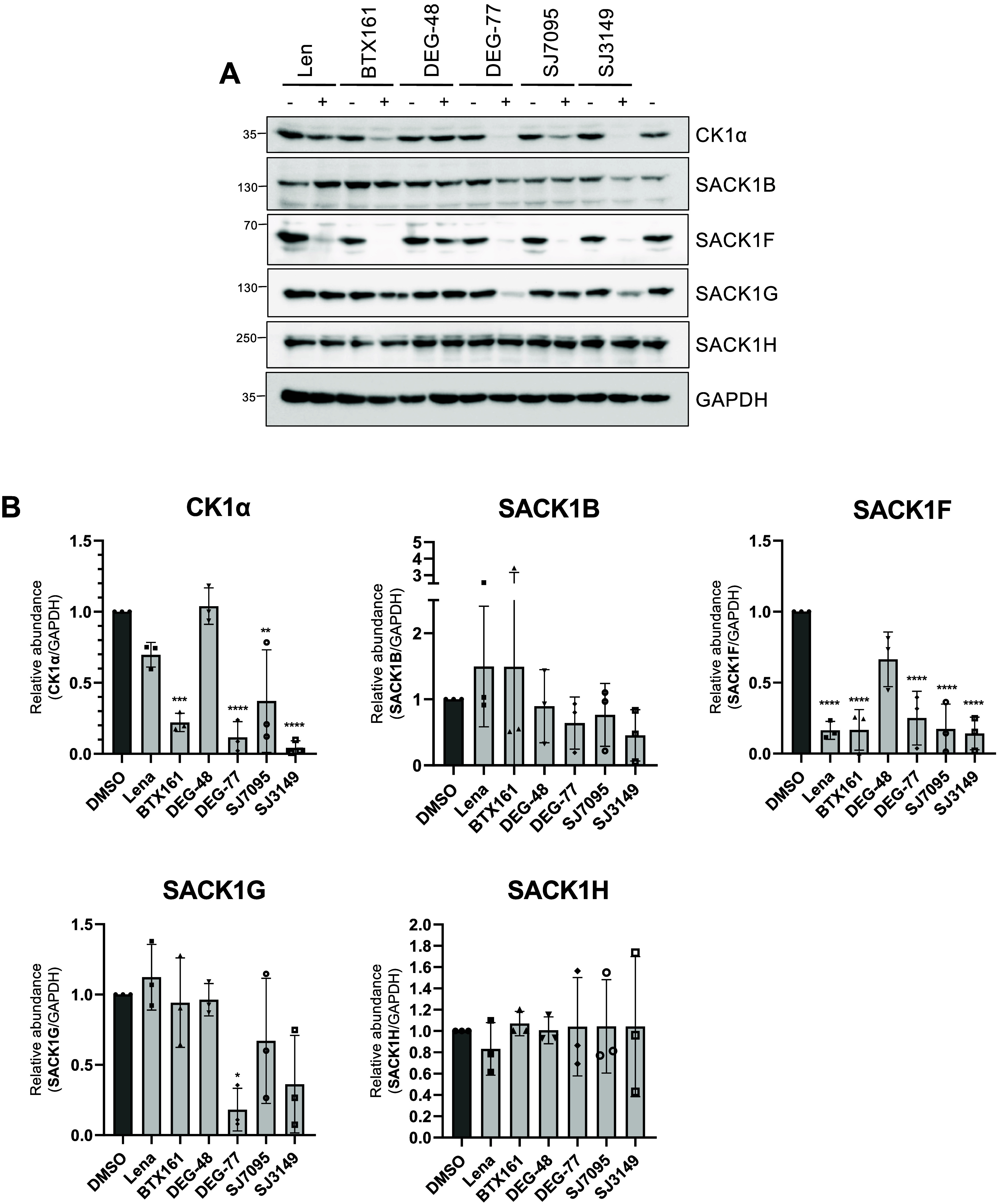
Comparison of CK1α degraders for their ability to codeplete the interacting SACK1 proteins. (A) Wild-type DLD1 cells were incubated with different IMiD-derived CK1α degraders: lenalidomide (10 μM), BTX161 (10 μM), DEG-48 (100 nM), DEG-77 (100 nM), SJ7095 (1 μM), and SJ3149 (100 nM) or DMSO control for 24 h before cell lysis. 20 μg of extract protein was resolved by SDS-PAGE, transferred to PVDF membrane, and analyzed by immunoblotting using the indicated antibodies. Blots are representative of at least 3 biological replicates. (B) Quantification by densitometry of protein signals shown in (A) using Fiji 1.53q (ImageJ). Each graph depicts the abundance of CK1α or SACK1 proteins normalized to GAPDH loading control relative to DMSO treatment control (*n* = 3, error bars represent mean ± SD). Statistical significance was determined using ordinary one-way ANOVA for comparing the means of > 2 groups to the DMSO group. The statistical significance is denoted on graphs. * *P* < 0.05, **P < 0.01, and ****P < 0.0001 compared with DMSO treatment.

### The Degradation of SACK1 Proteins by DEG-77 and SJ3149 Requires CK1α, CRBN, and the Proteasome

To examine if the observed codepletion of CK1α-SACK1 complexes by IMiDs is mediated through the recruitment of CK1α to CRBN (Figure 5A), we generated CSNK1A1^–/–^ (Figure S2) and CRBN^–/–^ ^31^ DLD1 cells by CRISPR/Cas9 genome editing (Figure 5B,C). Wild-type, CRBN^–/–^ and CSNK1A1^–/–^ DLD1 cells were treated with 100 nM DEG-77 (Figure 5B) or SJ3149 (Figure 5C) for 6 h. By immunoblotting, no CK1α protein was detected in CSNK1A1^–/–^ cells compared to both wild-type and CRBN^–/–^ DLD1 cells. Similarly, in comparison to levels detected in WT and CSNK1A1^–/–^ DLD1 cells, CRBN levels were almost absent in CRBN^–/–^ DLD1 cells, although a slight residual signal remained (Figure 5B,C). As expected, the degradation of CK1α by DEG-77 (Figure 5B) and SJ3149 (Figure 5C) observed in WT DLD1 cells was rescued in CRBN^–/–^ DLD1 cells. Excitingly, the depletion in levels of SACK1B, SACK1F, and SACK1G caused by DEG-77 (Figure 5B) and SJ3149 (Figure 5C) observed in WT DLD1 cells was completely rescued in both CRBN^–/–^ and CSNK1A1^–/–^ DLD1 cells, suggesting that the IMiD-induced depletion in levels of SACK1 proteins requires both CRBN and CK1α. We observed decreased levels of SACK1G in CSNK1A1^–/–^ DLD1 cells compared to WT or CRBN^–/–^ cells (Figure 5B,C). This is consistent with our previous observations where interaction between SACK1G and CK1α is essential for regulating the stability of each protein and depleting one or disrupting their interaction destabilizes the other.^12,14,16^

**Figure 5:**
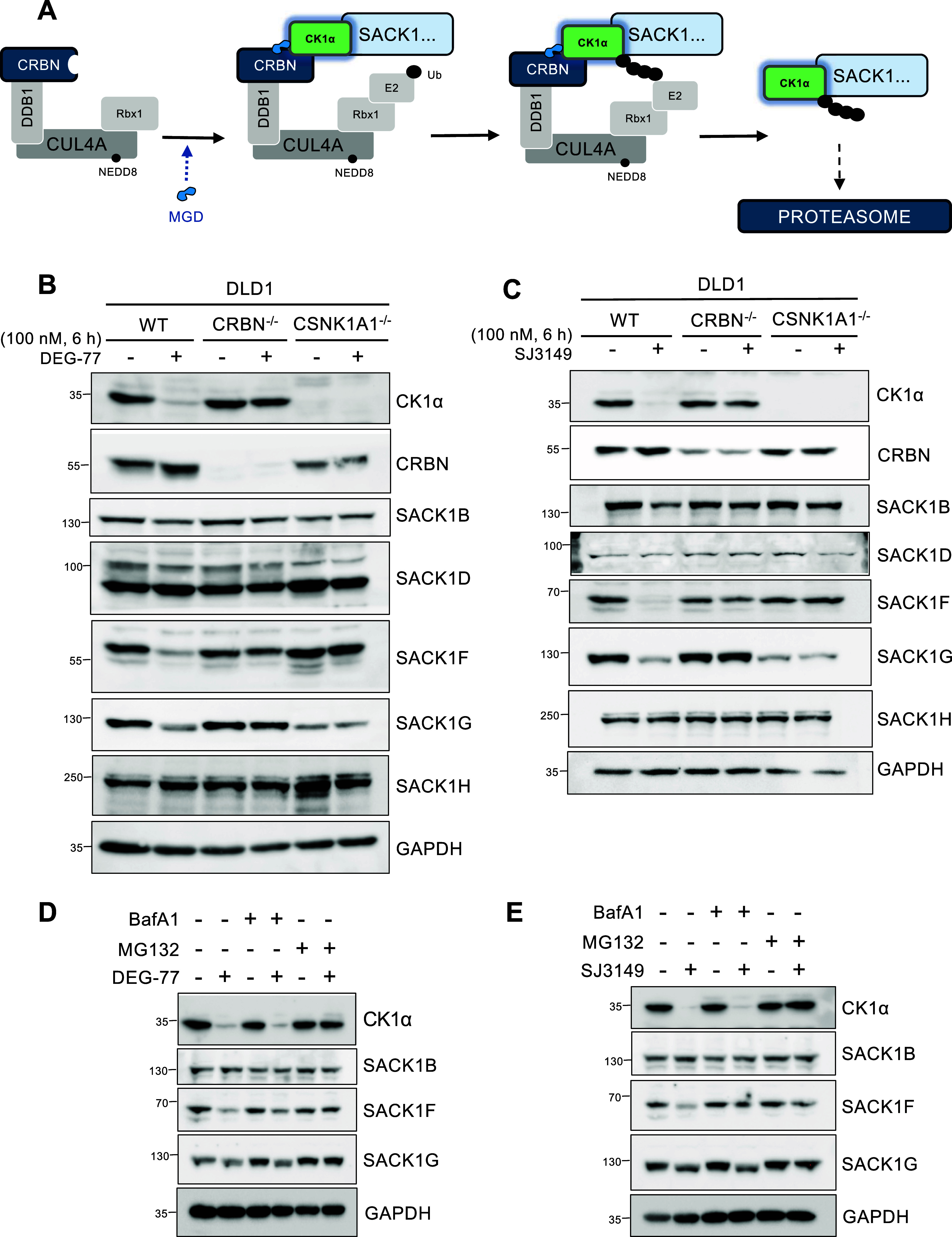
Degradation of SACK1 proteins by IMiDs DEG-77 and SJ3149 requires CK1α, CRBN, and the proteasome. (A) The postulated mechanism of action by which IMiD-derived molecular glue degraders (MGDs) DEG-77 and SJ3149 induce CK1α-SACK1 codegradation. By binding to substrate receptor cereblon (CRBN), both MGDs primarily recruit CK1α to CUL4^CRBN^ E3 ligases, and, as CK1α exists in complex with SACK1 proteins, it corecruits SACK1 proteins to the CUL4^CRBN^ E3 ligase complex. Depending on the proximity of the recruited CK1α-SACK1 complex to the CUL4^CRBN^ E3 catalytic site, specific CK1α-SACK1 complexes are potentially ubiquitinated and targeted for degradation via the proteasome. (B, C) Wild-type, CRBN^–/–^ and CSNK1A1^–/–^ DLD1 cells were incubated with 100 nM DEG-77 or DMSO (B) or 100 nM SJ3149 or DMSO (C) for 6 h before cell lysis. 20 μg of extract protein was resolved by SDS-PAGE and transferred to PVDF membrane for immunoblotting analysis. (D, E) As in (B), except wild-type DLD1 cells were pretreated with either MG132 (20 μM) or bafilomycin A1 (50 nM) for 2 h before treatment with DEG-77 (100 nM) or DMSO (D) or SJ3149 (100 nM) or DMSO (E) for a further 4 h before lysis. For (B–E), blots are representative of at least 3 biological replicates.

To determine how CK1α-SACK1 codegradation was mediated by DEG-77 and SJ3149, wild-type DLD1 cells were pretreated with either the proteasomal inhibitor MG132 (20 μM) or autophagy-lysosomal inhibitor bafilomycin A1 (50 nM) for 2 h prior to treatment with DEG-77 and SJ3149 (Figure 5D,E). The depletion in levels of CK1α, SACK1B, SACK1F, or SACK1G by DEG-77 and SJ3149 was completely rescued when cells were pretreated with MG132 but not bafilomycin A1 (Figure 5D,E), indicating that CK1α-SACK1 complex codegradation by DEG-77 and SJ3149 treatment is mediated by the proteasome.

To determine if we could detect enhanced ubiquitination of CK1α-SACK1 complexes upon DEG-77 treatment in the presence of MG-132, we employed homozygous ^*GFP/GFP*^ *SACK1F* knockin DLD1 cells^10^ and treated them with DMSO or DEG-77 in the presence or absence of MG-132. As expected, DEG-77 induced a robust degradation of CK1α and GFP-SACK1F, while MG-132 rescued their degradation (Figure S4). GFP-SACK1F IPs robustly coprecipitated CK1α (Figure S4), but under these conditions, only a slight, if any, increase in higher molecular weight signal was visible for GFP-SACK1F in the presence of both DEG-77 and MG-132 treatments compared to DMSO, MG-132, or DEG-77 controls alone (Figure S4). No higher molecular weight species for CK1α were visible in extracts or GFP-SACK1F IPs under any conditions (Figure S4). IP/IB assays to capture endogenous proteins are often not sensitive enough to capture polyubiquitinated proteins, especially because the heterogeneous nature of polyubiquitination can lead to molecular shifts of various sizes thereby diluting epitopes available for recognition by protein- or ubiquitin-specific antibodies. Additionally, given that MG-132 rescues degradation of the native molecular weight species of CK1α and GFP-SACK1F, it implies that deubiquitylases are potentially cleaving accumulated polyubiquitinated species. To circumvent these technical caveats of IPs and potentially map DEG-77-induced ubiquitination sites on endogenous CK1α and associated SACK1 proteins, we instead undertook an unbiased ubiquitin (diGly)-proteomics on DMSO vs DEG-77-treated DLD1 cell extracts (Figure S5A). To ensure we captured DEG-77-induced ubiquitinated peptides, we undertook a short 15 min course of DEG-77 when no substantial CK1α degradation occurs. Under these conditions, we found that upon DEG-77 treatment, enhanced ubiquitination on six Lys residues on CK1α (K65, K225, K152, K162, K16 and K304), two on SACK1G (K353 and K342) and two on SACK1B (K338 and K385) was observed compared to DMSO-treated controls (Figure S5B). Although the ubiquitination of Lys residues on SACK1G and SACK1B did not meet the significant threshold, the trends showing enhanced ubiquitination following DEG-77 treatment compared to DMSO controls show that DEG-77 leads to ubiquitination of these proteins. We avoided MG-132 treatment to preserve ubiquitinated residues upon DEG-77 treatment because MG-132 enhances ubiquitinated residues on proteins that also control their natural turnover, and this can compound data analysis on degrader-induced ubiquitin mapping.^47^

### CK1α-SACK1G Codegradation Mediated by DEG-77 Requires a Direct Interaction between CK1α and SACK1G

We have so far shown that the degradation of SACK1 proteins by lenalidomide-derived degraders requires CK1α to be present in cells. To definitively demonstrate that it is the interaction between CK1α and SACK1 proteins that facilitates their codegradation at the endogenous level, we employed skin fibroblast cells derived from a palmoplantar keratoderma (PPK) patient harboring the homozygous SACK1G^R265P^ mutation that does not interact with CK1α.^14^ Skin fibroblasts derived from an age- and sex-matched patient with no PPK pathogenesis served as a wild-type control. Loss of CK1α interaction and subsequent inhibition of WNT signaling underlies the pathogenesis of PPK caused by this SACK1G^R265P^ mutation (Figure 6A).^14^ Fibroblasts from a PPK patient (FIB04), or those from a wild-type control (FIB03) were treated with potent CK1α degraders DEG-35 and DEG-77, as well as a weaker degrader DEG-48, for 24 h (Figure 6B). As seen with other cell types before, both DEG-35 and DEG-77 caused a robust degradation of CK1α in PPK patient FIB04 as well as FIB03 control fibroblasts, while DEG-48 treatment did not cause any degradation of CK1α in any cells (Figure 6B). In FIB03 control fibroblasts, both DEG-35 and DEG-77 caused a robust depletion in levels of SACK1G compared to DMSO control (Figure 6B). However, despite DEG-35 and DEG-77 causing efficient degradation of CK1α in PPK FIB04 fibroblasts, there was no measurable codepletion of SACK1G^R265P^ (Figure 6B), indicating that the loss of CK1α binding protects SACK1G^R265P^ mutant from codegradation by IMiDs. In contrast, in both PPK patient FIB04 and control fibroblast FIB03 cells, DEG-35 and DEG-77 caused a reduction in levels of hyperphosphorylated SACK1D (Figure 6B), suggesting that the CK1α-interacting SACK1 proteins in these cells are potentially still susceptible to codegradation. The expression of SACK1F and SACK1B could not be detected in these fibroblast cells. To consolidate these findings further, we restored the stable expression of SACK1G-GFP or SACK1G^R265P^-GFP in SACK1G^–/–^ DLD1 cells.^10^ Treatment of these cells with DEG-77 caused a robust reduction in levels of CK1α and SACK1F (Figure 6C). In contrast, while DEG-77 caused a robust reduction in levels of SACK1G-GFP restored in SACK1G^–/–^ DLD1 cells, it failed to deplete SACK1G^R265P^-GFP (Figure 6C). Collectively, these results suggest that SACK1 proteins are only codegraded when recruited to CUL4A^CRBN^ E3 ligases via their association with CK1α.

**Figure 6:**
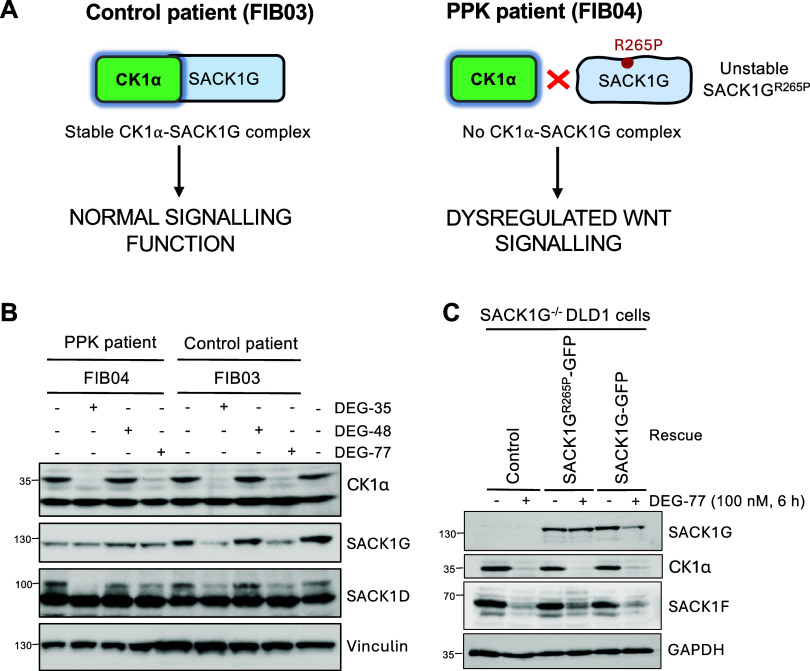
CK1α-SACK1 codegradation mediated by DEG-77 and SJ3149 requires a direct interaction between CK1α and SACK1 proteins. (A) Depiction of CK1α and SACK1G interaction that drives WNT signaling in normal cells (as in control fibroblasts, FIB03) and how this becomes disrupted with the pathogenic SACK1G^R265P^ mutation identified in a patient with palmoplantar keratoderma (as in PPK patient fibroblasts, FIB04). (B) Patient-derived fibroblast cells (FIB04 represents the PPK patient cells harboring the pathogenic SACK1G^R265P^ variant, while FIB03 represents an age and sex-matched control cell line) were treated with 100 nM of DEG-35, DEG-48, DEG-77, or DMSO for 24 h before cell lysis. 10 μg of extract protein was resolved by SDS-PAGE and transferred to PVDF membrane for immunoblotting analysis. (C) SACK1G^–/–^ DLD1 cells and those retrovirally transduced to stably express either SACK1G-GFP or SACK1G^R265P^-GFP were treated with DEG-77 (100 nM) or DMSO for 6 h before lysis. 20 μg of extract protein was resolved by SDS-PAGE and transferred to PVDF membrane for immunoblotting analysis. For (B, C), blots are representative of at least 3 biological replicates.

## Discussion

Lenalidomide-derived DEG-77 ^37^ and SJ3149 ^40^ are highly potent CK1α degraders. CK1α in cells is localized to unique subcellular compartments via its interaction with one of the eight SACK1 proteins (A–H).^11,13^ Here, we report that DEG-77 and SJ3149 not only degrade CK1α in multiple cell lines but also efficiently codeplete SACK1F, SACK1G and mitotic SACK1D, with similar potency and kinetics, while some codepletion of SACK1B was also observed. In contrast, lenalidomide induced poor degradation of CK1α, SACK1B, SACK1D, SACK1G, and SACK1H but degraded SACK1F completely, confirming the robust degradation of only CK1α-SACK1F complex.^31^ The depletion of CK1α and the associated SACK1 proteins caused by DEG-77 and SJ3149 was prevented in CRBN^–/–^ cells and by proteasome inhibitor MG132, suggesting CUL4A^CRBN^ E3 ligase-mediated polyubiquitination and subsequent degradation by the proteasome. Critically, SACK1 protein depletion by DEG-77 and SJ3149 was completely abolished in CSNK1A1^–/–^ cells, confirming an essential role for CK1α in the codestabilization of its interacting partners. The requirement for CK1α-SACK1G interaction for DEG-77-induced codegradation was further validated in cells derived from palmoplantar keratoderma patients harboring the CK1α-binding-deficient SACK1G^R265P^ mutation, where DEG-77 completely failed to degrade SACK1G^R265P^ while still degrading CK1α.

While definitive proof that DEG-77 and SJ3149 induce codegradation of CK1α-SACK1A–H complexes requires demonstrating direct ubiquitination of CK1α and associated SACK1 proteins by the CUL4A^CRBN^ E3 ligase machinery, substantial evidence supports codegradation, particularly for SACK1F. Despite genetic ablation of CK1α stabilizing SACK1F in DLD1 cells, all IMiD compounds that degrade CK1α to varying levels consistently deplete SACK1F levels. This suggests that the membrane-localized CK1α-SACK1F complex forms a productive ternary complex with CUL4A^CRBN^ E3 ligase upon IMiD treatment, leading to degradation rather than stabilization. The differential targeting of SACK1 proteins may explain the varied potency of IMiDs against CK1α degradation. For instance, lenalidomide’s weaker potency in degrading CK1α could be explained by failure of certain CK1α-SACK1 complexes to form productive ternary complexes with CUL4A^CRBN^. Indeed, SACK1G, though not degraded by lenalidomide, was identified as the most abundant interactor of CRBN when cells were exposed to lenalidomide.^25^ Further supporting this notion, overexpression of SACK1G in cells attenuates the ability of lenalidomide to degrade CK1α-SACK1F complexes.^31^ The observed difference in degradation kinetics between CK1α and different SACK1 proteins may also be explained by the inherent differences in the formation of CK1α-SACK1 complexes having an influence on their positioning toward CUL4A^CRBN^ E3 ligase and subsequent ubiquitination.

The codegradation of CK1α-SACK1G and CK1α-SACK1D complexes by MGDs requires careful interpretation, unlike that of SACK1F. The stability of CK1α and SACK1G proteins is codependent. Therefore, degradation of either would lead to destabilization of both. The mechanisms underlying this costability remain unresolved. Interpreting SACK1D codegradation is also complicated. SACK1D transcription and protein abundance are enhanced in mitosis, where it exclusively interacts with CK1α. This interaction results in SACK1D hyperphosphorylation, which is observed as an upward 25 kDa mobility shift on immunoblots.^6^ Although the mitotic hyperphosphorylated SACK1D disappears upon DEG-77 treatment, this could result from one of three possibilities: (i) degradation of CK1α prior to mitosis leaves no CK1α for mitotic SACK1D phosphorylation; (ii) the degraders inhibit cells from entering mitosis, which prevents SACK1D from binding CK1α; and (iii) CK1α-SACK1D complexes form in mitosis and are then codegraded. To definitively investigate codegradation, future experiments should involve treating cells arrested in mitosis with DEG-77, provided CUL4A^CRBN^ E3 ligase activity is maintained under these conditions.

The glutarimide ring of the IMiD defines IMiD-CRBN interactions, whereas the exposed phthalimide group mediates binding to the neo-substrates, dictating the nature of the resulting ternary complex.^48^ Rational design of the DEG series, involving differential additions to the phthalimide amine, yielded compounds with a range of CK1α degradation capabilities^38^ (Figure S1). Intriguingly, SJ3149, which achieved similar or better degradation of CK1α compared with DEG-77, degraded SACK1 proteins less efficiently. SJ7095, SJ0040, and SJ3149 were also derived from lenalidomide by additions to the amine group of the phthalimide moiety (Figure S1). However, the structural differences between SJ3149 and DEG-77 may influence the recruitment of specific CK1α-SACK1 complexes to CRBN and therefore produce slight structural differences in the resulting ternary complex, potentially accounting for the observed differences in the codegradation of the SACK1 proteins. While structures of some compounds with CRBN and CK1α have been reported,^35,39,48^ there are currently no structures that also include CK1α with the interacting SACK1A-H proteins.

Because many proteins in cells exist in macromolecular complexes, often defined by post-translational modifications, how degraders recruit the POI macromolecular complex and present the POI or the interacting partner surface to the E3 ligase determines whether the POI or the interactor is ubiquitinated and degraded. Here, we provide evidence of “*bystander degradation*”, where both CK1α and the interacting SACK1B, SACK1D, SACK1F and SACK1G proteins are robustly degraded by DEG-77 and SJ3149. Selective targeting of CK1α catalytic activity has been a major challenge in therapeutics against CK1α-driven malignancies, in part because of the pleiotropic roles of CK1α and the similarity of its kinase domain with other CK1 family members.^5^ SACK1 proteins provide a subcellular and functional context to CK1 isoforms and therefore provide opportunities by which specific CK1 functions can be selectively targeted. Although the DEG and SJ series of compounds assessed here did not display any selectivity toward specific CK1α-SACK1 complexes, they show that complexes other than CK1α-SACK1F can be targeted using MGDs and, in addition to lenalidomide, selective degraders of these other complexes may be achievable. An alternative approach using PROTACs directed toward individual SACK1 proteins may also be explored as a viable strategy of selectively targeting CK1α function without affecting its other pleiotropic roles.

Our work demonstrating differential CK1α-SACK1 degradation by different MGDs highlights the importance of protein and cellular context to TPD. SACK1­(A–H) are considered the scaffold-like molecular bridges that deliver CK1 isoforms to their substrates. This type of regulation is inherent to many multiprotein assemblies that regulate almost all cellular processes. Given the proximity-inducing nature of small molecule degraders and the role of protein–protein interactions in TPD evidenced here, the underappreciated aspect of bystander degradation, where neighboring proteins that form a complex with the POI are also targeted for degradation, provides both challenges and opportunities in developing targeted protein degraders.

## Materials and Methods

### Materials

The following tables (Tables 1–5) list materials employed in the study along with their sources and catalogue numbers.

**Table 1:** Cell Lines Used in This Study

**cell type**	**species**	**source**
wild-type DLD1	human	ATCC (CCL-221)
wild-type U2OS	human	ATCC (HTB-96)
wild-type A549	human	ATCC (CCL-185)
wild-type TOV-21G	human	ATCC (CRL-3577)
CRBN^–/–^ DLD1	human	^31^
CSNK1A1^–/–^ DLD1	human	this paper
SACK1G^–/–^ DLD1	human	^31^
FIB03	human	^14^
FIB04	human	^14^

**2:** Antibodies Used in This Study

**antibody**	**use**	**source**	**cat no.**	**species**	**conditions**
CK1α	IB/IP	MRC PPU Reagents and Services	SA527	sheep	1:1000 in 5% milk + TBS-T (IB) 1 μg/mg protein (IP)
SACK1B	IB	MRC PPU Reagents and Services	SA270	sheep	1:1000 in 5% milk + TBS-T
SACK1D	IB	MRC PPU Reagents and Services	SA102	sheep	1:1000 in 5% milk + TBS-T
SACK1F	IB	MRC PPU Reagents and Services	SA103	sheep	1:1000 in 5% milk + TBS-T
SACK1G	IB	Abcam	Ab121750	rabbit	1:1000 in 5% BSA + TBS-T
SACK1H	IB	MRC PPU Reagents and Services	SA273	sheep	1:1000 in 5% milk + TBS-T
CRBN	IB	CST	71810	rabbit	1:1000 in 5% BSA + TBS-T
GAPDH	IB	Proteintech	HRP-60004	mouse	1:5000 in 5% milk + TBS-T
Vinculin	IB	Abcam	Ab129002	rabbit	1:1000 in 5% BSA + TBS-T
IgG control	IP	MRC PPU Reagents and Services	SPI166	sheep	1 μg/mg protein

**3:** Plasmids Used in This Study

**protein expressed**	**vector**	**source**	**identifier**
SACK1G-GFP	pBabeD	MRC PPU Reagents and Services	DU29088
SACK1G^R265P^-GFP	pBabeD	MRC PPU Reagents and Services	DU71791
empty vector	pBabeD	MRC PPU Reagents and Services	DU33769

**4:** Compounds Used in This Study

**compound**	**source**	**cat no.**	**purity**	**conditions**
DEG-35	^38^	N/A	>95%	100 nM
DEG-54			>95%	100 nM
DEG-72			>95%	100 nM
DEG-48			>95%	100 nM
DEG-64			>95%	100 nM
DEG-61			>95%	100 nM
DEG-77			>95%	1–1000 nM
**compound**	**source**	**cat no.**		**conditions**
SJ7095	^40^	N/A		0.001–10 μM
SJ0040				0.001–10 μM
SJ3149				0.001–10 μM
MG132	Abcam	Ab141003		20 μM
Bafilomycin A1	Enzo Life Sciences	BML-CM110		50 nM
Lenalidomide	Cayman Chemicals	CAY14643		10 μM
BTX161	MRC PPU Reagents and Services	N/A		10 μM

**Table 5:** RT-qPCR Primers Used in This Study

**gene name**	**nucleotide sequence (5′ to 3′)**	**PrimerBank ID**	**source**
*β-actin*	**F:** CATGTACGTTGCTATCCAGGC	4501885a1	Millipore Sigma
	**R:** CTCCTTAATGTCACGCACGAT		
*CSNK1A1*	**F:** AGTGGCAGTGAAGCTAGAATCT	68303571c1	Millipore Sigma
	**R:** CGCCCAATACCCATTAGGAAGTT		
*SACK1A*	**F:** GGAGATGTGTGACAAAGTCCAG	46255016c3	Millipore Sigma
	**R:** CCAGCGAATTTCCTGCCTG		
*SACK1B*	**F:** TTGTCCAGGAACGAGTTTCAGA	61676088c2	Millipore Sigma
	**R:** AGGAATCATCAGTACCATGTGCT		
*SACK1C*	**F:** GGTGGTGATTGCAGTGATACG	291167743c3	Millipore Sigma
	**R:** GACAGTTGGCGATGTAGGGAG		
*SACK1D*	**F:** GGGAAGGTTCACGAAAAGTTCA	116235447c3	Millipore Sigma
	**R:** GACTGGGCATACAGGATTCGG		
*SACK1E*	**F:** AGAAACCCTCGGTCATAGGTG	153251791c2	Millipore Sigma
	**R:** CGCTGCACACTCCTTAGAATG		
*SACK1F*	**F:** CGTCGGCTTCTACATGCCC	156564371c1	Millipore Sigma
	**R:** GCTCTACGTTCTGTCCTGTCA		
*SACK1G*	**F:** TCGTGGATGAGAGTAACGTCA	115392149c1	Millipore Sigma
	**R:** CCAGGGCACCCTTGAACTTG		
*SACK1H*	**F:** CCAGGTGCTCCATAATGAGTCA	157311634c3	Millipore Sigma
	**R:** AATCCTGTAGTTGGACTTCCTCT		

## Methods

### Mammalian Cell Culture

Cells were cultured in either Dulbecco’s modified Eagle medium (DMEM) (DLD1, U2OS, A549) or Rosewell Park Memorial Institute (RPMI) 1640 medium (TOV-21G) supplemented with 10% (v/v) fetal bovine serum (FBS), except FIB04 and FIB03 fibroblasts, which were maintained in DMEM + 20% FBS. Media were also supplemented with 2 mM L-glutamine (Lonza), 100 U/mL penicillin (Lonza), and 0.1 mg mL^–1^ streptomycin (Lonza) before use with all cell types. Cells were stored in a humidified incubator at 37 °C with 5% CO_2_ and handled under aseptic conditions within a laminar flow hood. For passaging, cells were washed in PBS and incubated with trypsin/EDTA at 37 °C until detached and transferred to fresh plates.

### Generation of *CSNK1A1* ^–/–^ DLD1 Cells by CRISPR/Cas9

All CRISPR/Cas9 technology procedures were performed using a dual guide nickase approach as described previously.^49–54^ CK1α knockout DLD1 cells were generated by targeting the *CSNK1A1* locus with sense guide RNA (pBabeD-puro vector, DU57522); GTCCAAGGCTGAATTCATTGT and antisense guide RNA (pX335-Cas9-D10A vector, DU57527); GATCCTGAGAGACGAAGCTGG. For transfection, 1 μg of each guide RNA was diluted in 1 mL of Opti-MeM (Gibco) and 20 μL of polyethylenimine (PEI: 1 mg mL^–1^). The transfection mixture was vortexed for 15 s and incubated for 20 min at RT before adding dropwise to a 10 cm diameter dish containing 70% confluent DLD1 cells in complete media. Media was replaced 24 h post-transfection with media containing 2 μg/ml puromycin and incubated for 48 h. Surviving cells were cultured in fresh complete medium until single cells were sorted and plated in individual wells of a 96-well plate, which was precoated with 1% (w/v) gelatin (Sigma) and contained complete conditioned media supplemented with 20% FCS. Viable clones were expanded and screened by immunoblotting for complete knockout. Only one CK1α knockout clone was identified, and this was then verified by DNA sequencing. Genomic DNA was isolated using DNeasy Blood & Tissue kit (69505, Qiagen). Primers were generated to amplify the region surrounding the guide RNA target sites with the following primer pairs: CSNK1A1 exon 1 (Forward: AGTAACAGGTAACACCTGCTGAGC; Reverse: AGACATTAGCAAAACTCCAAGTCGC). The region was amplified by polymerase chain reaction (PCR) using KOD Hot Start Polymerase (Merck) according to the manufacturer’s instructions. The PCR products were visualized on a 1.5% agarose gel using SYBR Safe DNA gel stain (Invitrogen) and 1 kbp DNA ladders (Promega). The PCR products of positive clones were then cloned into competent cells using the CloneJET PCR Cloning Kit (Thermo Fisher Scientific) according to the manufacturer’s protocol. Isolation of plasmid DNA and sequencing was performed by the MRC PPU DNA sequencing and services (http://mrcppureagents.dundee.ac.uk).

### Treatment of Cells with Compounds

The following compounds in DMSO were added directly to the culture medium at the indicated concentrations and incubated with cells for the specified times before cell lysis: DEG compounds (35, 54, 52, 48, 64, 61, 77),^38^ St. Jude compounds (SJ7095, SJ0040, SJ3149),^40^ lenalidomide (Cayman Chemicals), BTX161 (MRC PPU Reagents and Services), MG132 (Abcam), and bafilomycin A1 (Enzo Life Sciences). An equivalent volume of DMSO was added to the cells as a treatment control.

### Retroviral Transduction

Retroviral vectors were generated by cotransfecting pBabe-puromycin vectors encoding the desired construct (6 μg), pCMV5-gag-pol (3.2 μg), and pCMV5-VSV-G (2.8 μg) (Cell Biolabs) into a 10 cm diameter dish of ∼70% confluent HEK293-FT cells as described previously.^14^ In short, plasmids were resuspended gently in 1 mL of Opti-MEM medium before being mixed with 24 μL of PEI (1 mg mL^–1^). The transfection mixture was incubated at RT for 20 min and then added dropwise to the HEK293-FT cells. Next day, the culture medium was replenished for 24 h before the retroviral medium was collected and passed through a 0.45 μm sterile syringe filter. Target cells (∼70% confluent) were transduced with the retroviral medium (typically at 1:1 ratio with normal cell culture medium) containing 8 μg/mL Polybrene (Sigma-Aldrich) for 24 h. After 24 h, the medium was replaced with fresh culture medium. The medium was again replaced the next day with fresh medium containing 2 μg/mL puromycin for the selection of transduced cells. Those cells that survived puromycin selection after all nontransduced control cells died completely by puromycin treatment were taken forward for subsequent experiments.

### Cell Lysis

Cells were harvested by removing culture media, washing twice with ice-cold PBS and then scraping directly into ice-cold NP-40 cell lysis buffer (50 mM Tris-HCl pH 7.5, 0.27 M sucrose, 150 mM NaCl, 1 mM EGTA, 1 mM EDTA, 1 mM sodium orthovanadate, 10 mM sodium b-glycerophosphate, 50 mM sodium fluoride, 5 mM sodium pyrophosphate and 1% NP-40) supplemented with 1× complete protease inhibitor cocktail (Roche), and 1 mM DTT. Cell extracts were transferred to Eppendorf tubes and incubated on ice for 10 min, then clarified by centrifugation at 14,000 rpm for 15 min at 4 °C. Protein supernatants were transferred to fresh tubes, and protein concentration was measured by Bradford assay as previously described.^55^ For SDS-PAGE analysis, samples were prepared to an equal concentration by normalizing with NP-40 lysis buffer and 1× LDS sample buffer supplemented with 2.5% β-mercaptoethanol. Samples were boiled at 95 °C for 5 min to allow for protein denaturation before loading.

### SDS-PAGE and Immunoblotting

Samples prepared in 1× LDS sample buffer containing equal amounts of protein (10–20 μg) were loaded into 10% polyacrylamide gels or 4–12% NuPAGE Midi Bis-Tris gels (Invitrogen) and resolved using 120 V for 90–120 min. Protein was then transferred to PVDF membrane, and successful transfer was verified by Ponceau S staining. Membranes were blocked in 5% (w/v) nonfat milk (Marvel) in TBS-T (50 mM Tris–HCl, pH 7.5, 150 mM NaCl, 0.2% Tween 20) and incubated overnight at 4 °C with the appropriate primary antibodies. After incubation, primary antibodies were removed, and membranes were washed using TBS-T before being subjected to a second incubation with HRP-conjugated secondary antibodies for 1 h at RT. Secondary antibodies were prepared in 5% nonfat milk + TBS-T with goat antirabbit IgG (7074, CST, 1:2500) or rabbit antisheep IgG (31,480, Thermo Fisher Scientific, 1:2500). Membranes were again washed after incubation and then exposed to an enhanced chemiluminescent (ECL) substrate for 1 min to generate an HRP-linked protein signal. Protein bands were visualized using the ChemiDoc Imaging System (Bio-Rad).

### Immunoprecipitation

Extract containing 1 mg of protein was incubated with 2 μg of antibody (either against CK1α or IgG control) or GFP-trap beads for 16 h on a rotating platform at 4 °C, while a portion of these extracts was retained as input. For antibody IPs, protein G Sepharose beads were equilibrated using ice-cold NP-40 lysis buffer (supplemented with 1 mM DTT) and prepared as a 50% slurry stock. 20 μL of bead slurry was added to each protein extract/antibody mix, and both were incubated together on a rotating platform for 1 h at 4̊C to capture antibody-bound proteins on beads. Beads were then pelleted by centrifugation (1200 rpm, 5 min), and supernatants were stored as flow-through extracts (FT). Beads were washed three times with 0.5 mL of ice-cold NP-40 lysis buffer before they were mixed in 50 μL of 1× LDS sample buffer. Immunoprecipitates (IPs) were released from the beads by boiling samples at 95̊C for 10 min. Input and IP samples were then resolved by SDS-PAGE, transferred to PVDF membrane, and immunoblotted for analysis.

### Quantification and Data Analysis

Numerical data representing immunoblot quantification performed by Fiji 1.53q (ImageJ) was processed using Microsoft Excel before fold change values were transferred to GraphPad Prism (v.9.4.0) for graph preparation. Quantification graphs are presented as scatter plots with bars and error bars representative of the mean ± standard deviation (s.d.) overlaid with individual datapoints from three biological replicates.

### RNA Generation and Quantitative PCR (qPCR) Analysis

TOV-21G and SCCOHT-1 cells were seeded at 1.2 × 10^6^ cells per condition in a six-well plate and treated with either DMSO or 100 nM DEG-77 in biological triplicate for 24 h. After incubation, cells were harvested via trypsinization and pelleted using centrifugation at 500*g* for 3 min. Pellets were washed once via resuspension in 1 mL of PBS, which was then removed after centrifugation. RNA extraction was performed using a Qiagen RNeasy Mini Kit (catalogue #74104) according to the manufacturer’s instructions. The concentration and purity of the resulting RNA extracts were estimated using a NanoDrop One^C^ Microvolume UV–vis Spectrophotometer (Thermo Fisher Scientific). RT-qPCR analysis was performed using the Luna Universal One-Step RT-qPCR Kit (New England Biolabs, catalogue #E3005) according to the manufacturer’s instructions with the following modifications: the final concentration of both forward and reverse primers was 0.5 μM. The primer sequences used are detailed in the Materials and Methods section. The following table (Table 6) describes the RT-qPCR settings specified on a Bio-Rad CFX Connect Real-Time PCR Detection System:

**Table 6:** RT-qPCR Conditions Employed

**step**	**time**	**temperature** (°C)	**repetitions**
reverse transcription	15 m	55	
initial activation	5 m	95	
denaturation	15 s	95	45 cycles
annealing	30 s	55	
extension + plate read	30 s	60	
melt curve generation	0.5 °C/5 s	55 to 95	

The relative mRNA level was calculated using the 2^(−ΔΔCt)^ method with β-actin as the reference gene. 2^(−ΔΔCq)^ values are presented as the mean ± standard error of the mean (SEM) overlaid with individual datapoints from three biological replicates. Δ*Cq* values were used to calculate statistical significance using Welch’s *t* test: *p < 0.05, **p < 0.01, ***p < 0.001, and ****p < 0.0001. Data was analyzed using Microsoft Excel (v16.78.3) and GraphPad Prism (v10.1.1).

### DiGly Ubiquitinomics Analysis

For diGly ubiquitinomics analysis, cells treated with DEG-77 or DMSO were subjected to anti-K-ε-GG immunoaffinity enrichment followed by LC–MS/MS analysis. WT DLD1 cells were treated with either DMSO or 100 nM DEG-77 for 15 min prior to lysis in urea lysis buffer (8 M urea in 50 mM triethylammonium bicarbonate [TEABC] supplemented with protease and phosphatase inhibitor cocktail tablets). Samples were then sonicated in an ultrasonic water bath at 4 °C for 10 min. Subsequent steps were done at RT to avoid carbamylation. Extracts were centrifuged at 12,000 rpm for 10 min. The supernatant was collected, its protein concentration measured using bicinchoninic acid (BCA) protein estimation assay, and samples reconstituted to 3 mg mL^–1^ protein concentration for subsequent in-solution digestion. For in-solution digestion and peptide cleanup, all steps were done at RT. Samples were reduced in 5 mM dithiothreitol (DTT) for 60 min on a shaker at 750 rpm, then alkylated using 10 mM chloroacetamide (CAA) for 20 min on the shaker at 750 rpm, in the dark. The extracts were then incubated with LysC for 4 h at 1:100 ratio of enzyme:extract protein (by mass). Following LysC digestion, the extracts were diluted with 50 mM TEABC to reduce the concentration of urea to < 2 M. Trypsin at a 1:50 ratio of enzyme:extract protein (by mass) was used for the in-solution digestion and incubated for 16 h. The reaction was quenched with the addition of trifluoroacetic acid (TFA) to give a final concentration of 1% TFA (v/v), and samples were desalted on 200 mg Sep-Pak C18 cartridges (Waters). For Sep-Pak cleanup, the following solvents were prepared fresh: activation solvent (100% [v/v] acetonitrile [ACN]); Solvent-1 (0.1% [v/v] TFA in ACN); Solvent-2 (0.1% [v/v] formic acid [FA] in ACN); Solvent-3 (50% [v/v] ACN in 0.1% [v/v] FA). Sep-Pak cartridges were each equilibrated with 5 mL activation solvent twice, followed by 5 mL of Solvent-1 twice, and finally with 5 mL of Solvent-2 twice. Samples were then loaded onto the equilibrated C18 cartridges, washed with 5 mL of 0.1% TFA four times, then with 5 mL of 0.1% FA once. Samples were then eluted with 6 mL of Solvent-3. Desalted samples were then dried completely in a SpeedVac concentrator.

For diGly peptide enrichment, magnetic beads conjugated to the anti-K-ε-GG antibody (PTMScan HS Ubiquitin/SUMO Remnant Motif Kit; Cell Signaling #59322) were washed thrice in 1 mL of ice-cold PBS and reconstituted in 200 μL of PBS. 5 μL magnetic beads were directly added to the peptide supernatant, obtained from dried digested peptides which were reconstituted in 1 mL of immunoaffinity purification (IAP) buffer by repeat pipetting and clarified by centrifugation at 10,000*g* for 5 min at 4 °C. The magnetic anti-K-ε-GG antibody bead-peptide mix was incubated on an end-over-end rotating platform for 2 h at 4 °C. The tube was then placed in a magnetic stand for 10 s, and the unbound peptide solution was discarded. The magnetic beads were washed with 1 mL of IAP buffer thrice, followed by 2 washes with 1 mL of ice-cold Milli-Q water. The beads were then incubated with 0.15% TFA, and the diGly-modified peptides were collected. Peptide solutions were subjected to the stage-tip-based C18 cleanup. The peptides were dried in SpeedVac, then reconstituted in 0.1% FA. The peptides were passed through C18 stage tip that were activated by passing 30 μL of 100% ACN and equilibrated with 0.1% FA. The column was washed again twice with 0.1% FA, then the peptides were eluted in an Eppendorf tube by using 50% ACN/0.1% FA. The eluted peptides were dried in SpeedVac and stored in −80 °C until mass spectrometry analysis.

For ubiquitinome analysis, peptides were separated on an analytical column (Acclaim PepMap RSLC C18, 75 μm × 50 cm, 2 μm, 100 Å) at a flow rate of 800 nL/min, using a step gradient of 4–8% solvent B (90% ACN/0.1% FA) for the first 0.6 min, followed by 8–22.5% up to 18.6 min (22.7–35%), and up to 18.6–27.7 min. The total run time was set to 30 min. The data were acquired on an Orbitrap Astral in data-independent acquisition. For MS1, the resolution was 2,40,000, AGC target 50,00,000, scan range 380–980. The DIA isolation window used was 2, collision energy HCD 25%, AGC target 50,000, and DIA window type Auto.

The raw files were searched using DIA-NN, version 1.8 (https://github.com/vdemichev/DIaNN). Human UniProt was used as the protein sequence database. The following parameters were used for the analysis: 2 missed cleavages and a maximum of two variable modifications per peptide were allowed (acetylation of protein N-termini and oxidation of methionine). Carbamidomethylation of cysteines was set as a fixed modification, and K-GG was added. This data analysis was carried out using library-free analysis mode in DIA-NN with “deep-learning-based spectra and RTs prediction” enabled. MBR was enabled. Five biological replicates were acquired per condition; based on replicate-level quality control assessing CSNK1A1 diGly peptide recovery, two replicates per condition with suboptimal enrichment were excluded from downstream analysis. Statistical analysis was performed using the remaining three replicates per condition, with peptide intensities log_2_-transformed prior to analysis. Missing values were retained for peptides detected exclusively in DEG-77 samples and imputed at a lower detection limit for statistical testing. Differential ubiquitination was assessed using Welch’s *t* test, and results were visualized using volcano plots based on log_2_ fold change and nominal p-values.

## Supplementary Material



## Data Availability

The original data generated during this study are publicly available at Mendeley Data (https://data.mendeley.com/datasets/3zygrwcj4t/1).

## Notes

Chemical structures of all the compounds used in this study (Figure S1); experimental validation of the *CSNK1A1* ^*–/–*^ CRISPR/Cas9 genome editing (Figure S2); effect of DEG-77 on transcription of SACK1 proteins (Figure S3); assessment of DEG-77-induced ubiquitination of SACK1F and CK1α at the endogenous level (Figure S4); and unbiased global diGly ubiquitinomics analysis in DLD1 cells following DEG-77 treatment (Figure S5) (PDF)
