# Supplementary material for: The Contribution of Native Protein Complexes to Targeted Protein Degradation
Source: ACS Chem Biol. 2026 Apr 9;21(5):1095–111. doi: 10.1021/acschembio.6c00098 (PMC13184940; doi:10.1021/acschembio.6c00098)
Supplement: Supplementary file 1 [file cb6c00098_si_001.pdf]

The contribution of native protein complexes to targeted protein degradation.

Lorraine Glennie[1], Nicole Curnutt[2], Gajanan Sathe[1], Brune Le Chatelier[1], Freya Goff[1], Jin-Feng Zhao[1], Tyrell Cartwright[1], Karen Dunbar[1], Nicola T Wood[1], Thomas J Macartney[1], Christina M. Woo[2] and Gopal P. Sapkota[1*]

[1]Medical Research Council Phosphorylation & Ubiquitylation Unit (MRC-PPU), School of Life Sciences, University of Dundee, Dow Street, Dundee, DD1 5EH, Scotland, UK

[2]Department of Chemistry and Chemical Biology, Harvard University, Cambridge, MA 02138, USA and Broad Institute of MIT and Harvard, Cambridge, MA 02142, USA.

[*]Corresponding author: g.sapkota@dundee.ac.uk

Supporting Information: Figures and Legends

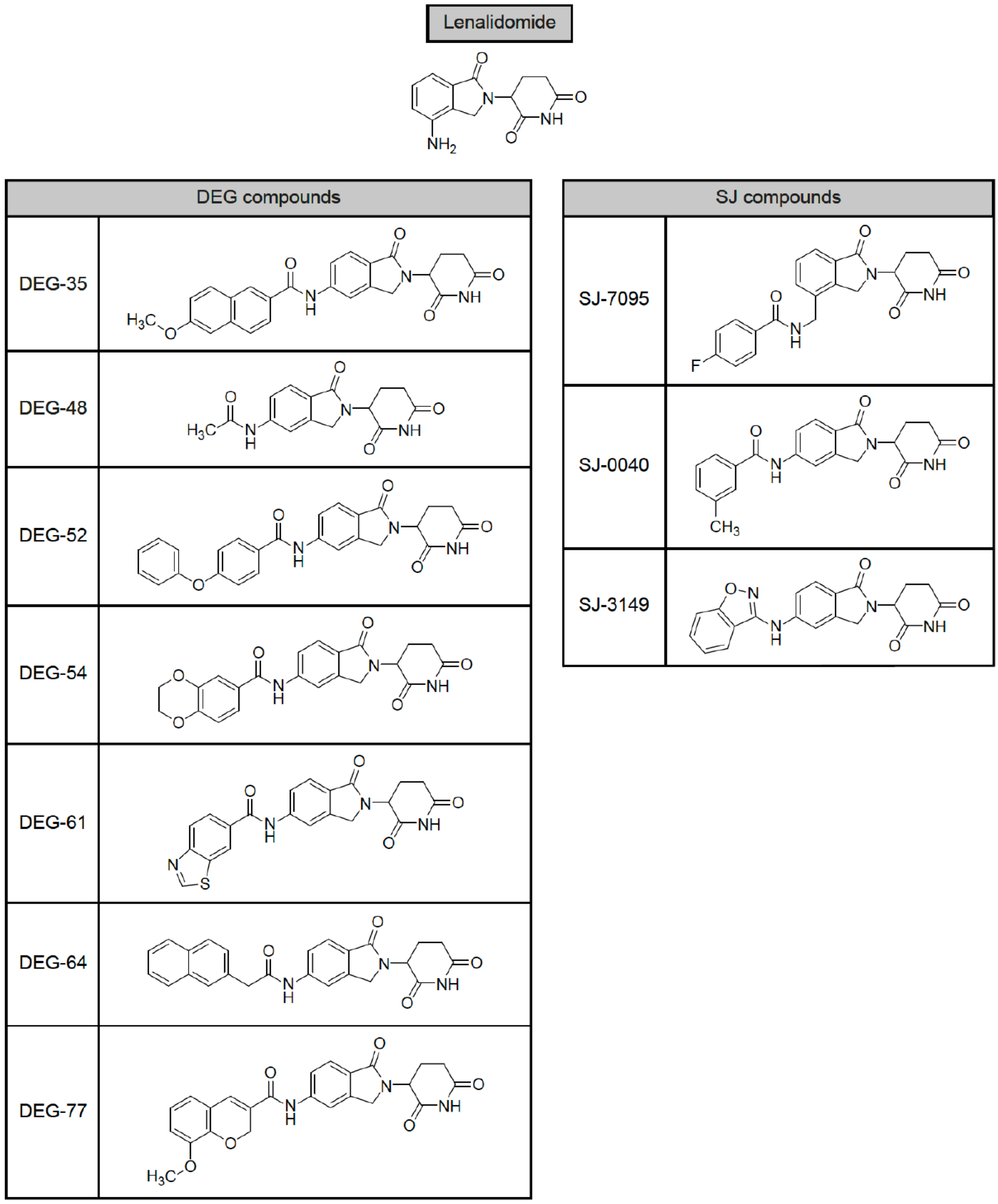

Figure S1

Figure S1: Structures of the DEG- and SJ-series of compounds derived from lenalidomide employed to assess their ability to co-degrade SACK1 domain-containing proteins along with CK1α. The structure of lenalidomide, from which all DEG and SJ compounds derive, is shown at the top. Below this, in the left-hand table, each DEG compound used in this study is listed with its unique chemical structure. The right-hand table depicts the structure of each SJ compound employed in this study.

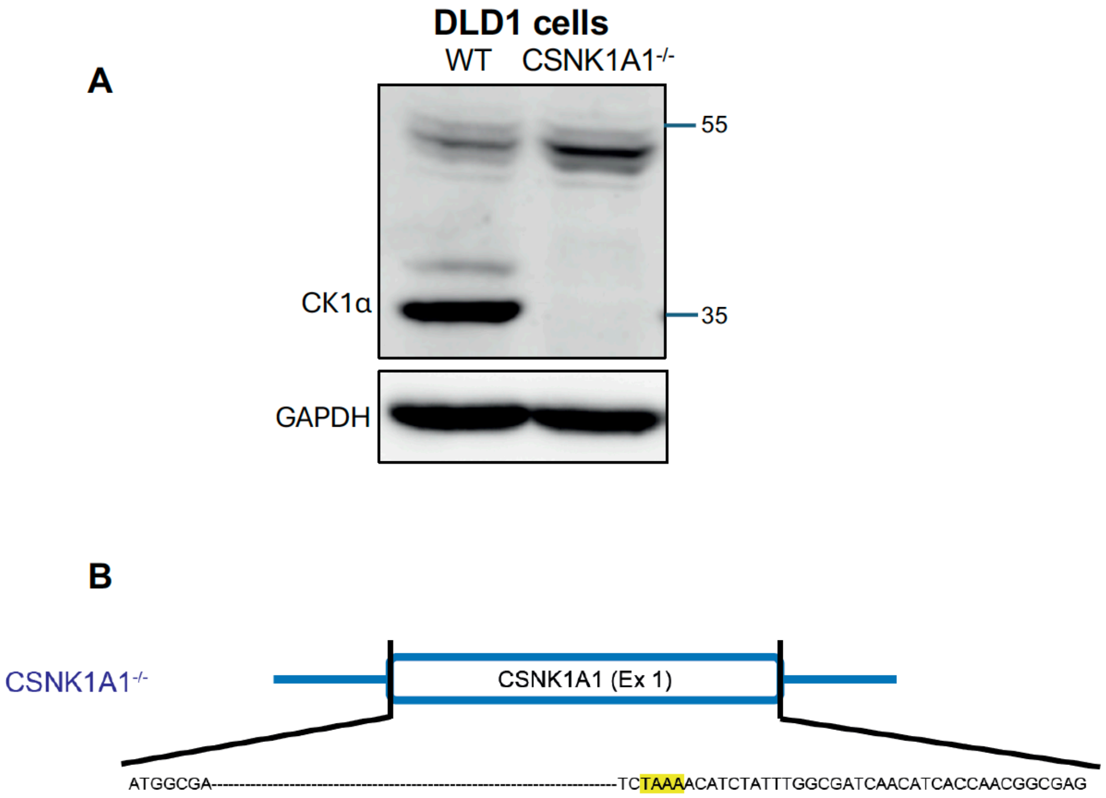

Figure S2

Figure S2: Confirmation of *CSNKA1*$^{-/-}$ DLD1 cells.

(A) Extracts (20 µg protein) from DLD1 wild-type (WT) and CSNK1A1$^{-/-}$ cells were resolved by SDS-PAGE and subjected to immunoblotting with the indicated antibodies. (B) DNA sequencing of CSNK1A1 (exon 1) confirming alterations in CSNK1A1$^{-/-}$ alleles. Nucleotide deletions and single nucleotide polymorphisms (highlighted nucleotides) predict premature stop codons.

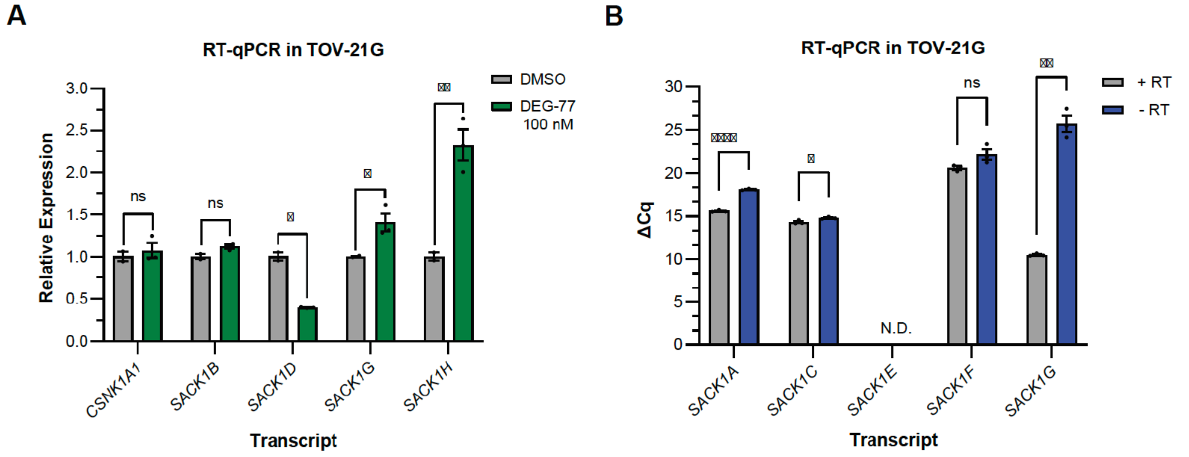

Figure S3: Effect of DEG-77 on transcription of SACK1 proteins.

(A) TOV-21G cells were treated with DMSO or 100 nM DEG-77 for 24 h prior to cell lysis, RNA isolation, cDNA synthesis and analysis of the indicated transcripts by RT-qPCR. The relative transcript expression is normalized to actin and the means of three biological replicates ± SEM are plotted. Welch's t test, *$p < 0.05$, **$p < 0.01$, ***$p < 0.001$, and ****$p < 0.0001$.

(B) As in (A) except that qPCR for the indicated genes was performed from DMSO-treated RNA preps with (+) or without (-) the addition of reverse transcriptase (RT) enzyme. The presence of mRNA corresponding to a gene, as seen for the *SACK1G* transcript, is indicated by a large and significant reduction in the ΔCq (actin-normalized Cycle quantification) value with the addition of RT enzyme. Conversely, little to no change in the ΔCq value in the presence of RT enzyme implies little to no mRNA is present in the sample and any observed Cq signal is potentially due to genomic DNA contamination. N.D. No Detection: no signal for the *SACK1E* transcript was detected in TOV-21G and in the SCCOHT-1 (-) RT samples. The means of three biological replicates ± SEM are plotted. Welch's t test, *$p < 0.05$, **$p < 0.01$, ***$p < 0.001$, and ****$p < 0.0001$.

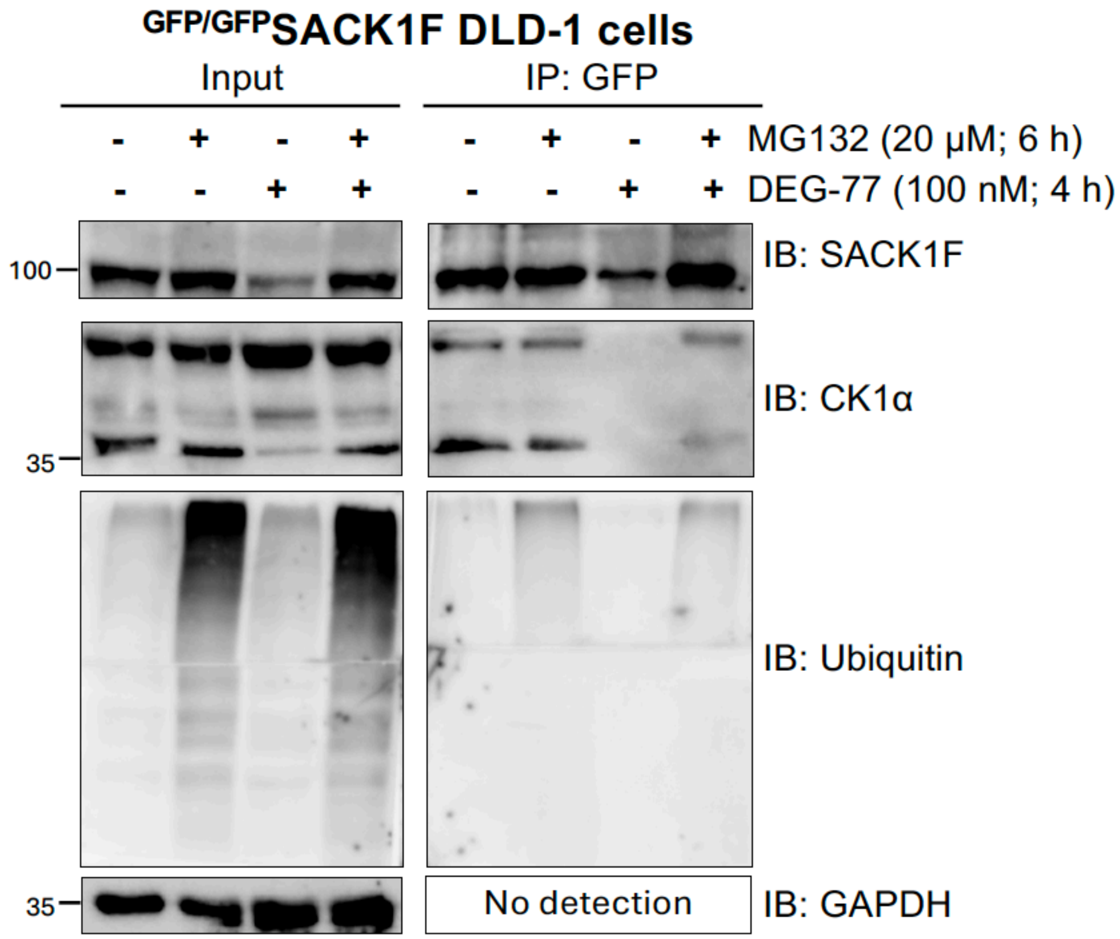

Figure S4: Assessment of DEG-77 induced ubiquitination of SACK1F and CK1α at the endogenous level. $^{GFP/GFP}$SACK1F homozygous KI DLD1 cells were treated with DMSO or MG-132 (20 μM) for 2 h followed by DMSO or DEG-77 (100 nM) for further 4 h prior to lysis as indicated. Extracts (1 mg protein) were subjected to immunoprecipitation with GFP-trap beads to immunoprecipitate (IP) GFP-SACK1F. Extracts (20 μg protein) or GFP-SACK1F IPs were resolved by SDS-PAGE and subjected to immunoblotting with the indicated antibodies.

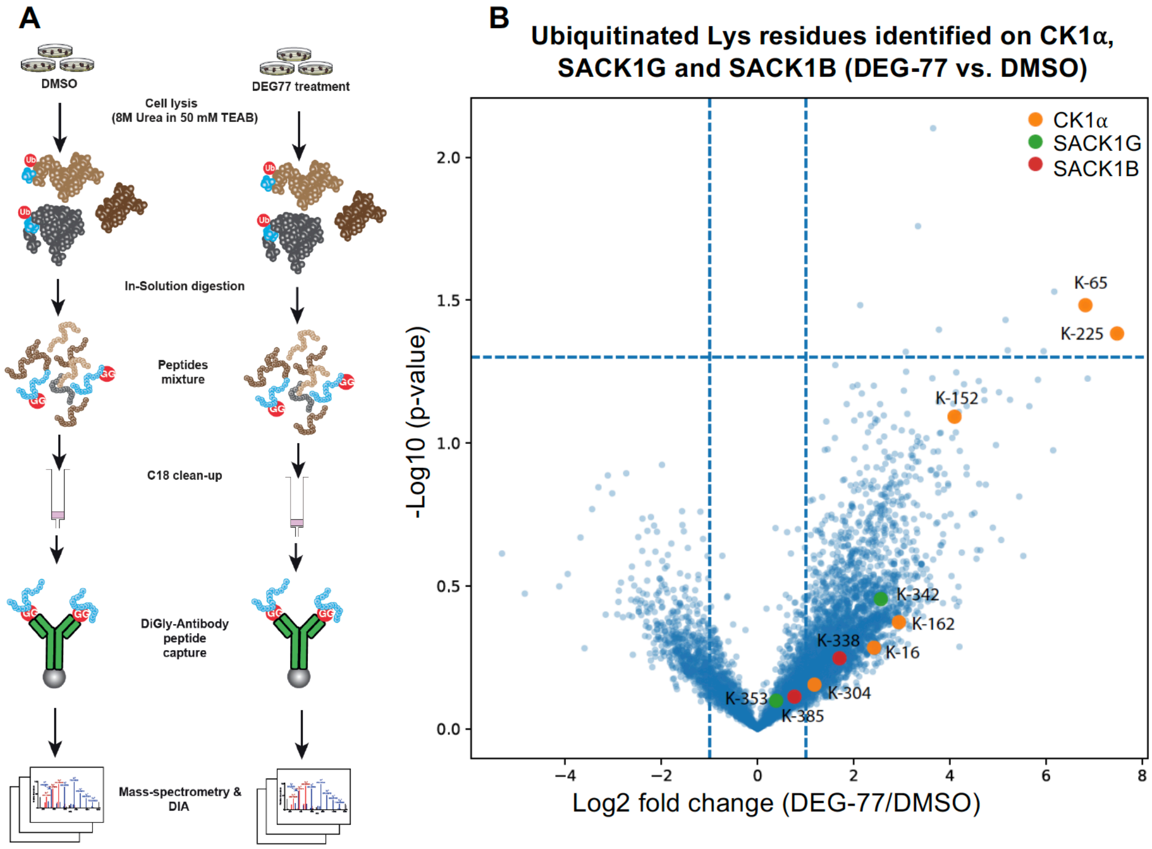

Figure S5: Unbiased global diGly ubiquitinomics analysis in DLD1 cells following DEG-77 treatment.

(A) DLD1 cells were treated with DMSO or 100 nM DEG-77 for 15 minutes prior to lysis. The schematic illustrates the key steps in sample preparation and diGly ubiquitinome profiling by mass spectrometry.

(B) Volcano plot showing changes in abundance of ubiquitinated peptide identified in DLD1 cells treated with DEG-77 compared to DMSO control. Indicated are the specific Lys residues on CK1α, SACK1G and SACK1B that were identified. While K-65 and K-225 on CK1α were found to be significantly ubiquitinated upon DEG-77 compared to DMSO treatment, the abundance of all other ubiquitinated peptides identified on CK1α, SACK1G and SACK1B was found to be enhanced upon DEG-77 treatment even though the increase was found not to be statistically significant. Data obtained from 3 independent biological replicates.
